# Total cholesterol, arterial stiffness, and systolic blood pressure: a mediation analysis

**DOI:** 10.1038/s41598-020-79368-x

**Published:** 2021-01-14

**Authors:** Haojia Chen, Youren Chen, Weiqiang Wu, Zefeng Cai, Zhichao Chen, Xiuzhu Yan, Shouling Wu

**Affiliations:** 1 Shantou University Medical College, Shantou, 515041 Guangdong China; 2 Department of Cardiology, First Hospital of Medical College of Shantou University, Shantou, 515041 Guangdong China; 3 Department of Cardiology, Second Affiliated Hospital of Shantou University Medical College, Shantou, 515041 Guangdong China; 4 School of Foreign Language, Guangdong Polytechnic Normal University, Guangzhou, 510000 Guangdong China; 5 Department of Cardiology, Kailuan General Hospital, North China University of Science and Technology, Tangshan, 063001 China

**Keywords:** Diseases, Health care, Risk factors

## Abstract

On the basis of 45,092 participants (mean age of 54.04 ± 13.09 years) from the Kailuan study, this study was performed to explore the relationships among total cholesterol (TC), brachial-ankle pulse wave velocity (BaPWV), and systolic blood pressure (SBP) and quantify their separate effects. The correlations among TC, SBP, and BaPWV were analyzed using multivariate linear regression models. Mediation analysis was performed to determine whether the effect of TC on SBP can be explained by arterial stiffness. Multivariate linear regression analysis showed that for every one standard deviation increase in TC and BaPWV, SBP increased by 0.33 mmHg and 0.044 mmHg, respectively; for every one standard deviation increase in TC, BaPWV increased by 5.34 cm/s. Mediation analysis showed that the TC-induced SBP elevation was mediated by arterial stiffness in more than half of the whole cohort (indirect effect, 0.73; percent mediated, 54.5%). Furthermore, the TC-induced SBP elevation was mediated by arterial stiffness in less than half of the males (indirect effect, 0.70; percent mediated, 47.9%); however, the results were not statistically significant in females. In conclusion, TC and BaPWV are positively correlated with SBP, whereas TC is positively correlated with BaPWV. Almost half of the increase in SBP contributed to TC is mediated by arterial stiffness.

## Introduction

Hypercholesterolemia and hypertension are the two most common risk factors for cardiovascular diseases and often co-occur^1,2^. Previous studies have demonstrated that the total cholesterol (TC) level is positively correlated with blood pressure in both the general population and in patients with hypertension^3,4^. However, the causal relationship between the TC level and blood pressure and the underlying mechanisms remain unclear. Recent studies have elucidated different mechanisms to explain the rise in blood pressure contributed to TC levels^5–8^. In particular, Borghi^6,7^ and Pereira^8^ found that the pathogenic effect of hypercholesterolemia on hypertension might be closely associated with the effect of a high cholesterol level on peripheral vascular tone and the role of tissue renin-angiotensin system. However, this hypothesis has not been tested in epidemiological studies.

Measurement of the brachial-ankle pulse wave velocity (BaPWV) is a noninvasive technique used to estimate the central and peripheral arterial stiffness and has been widely used in clinical settings because of its simplicity and high reproducibility. Meanwhile, a close relationship exists between arterial stiffness and vascular tone^9^. Research has confirmed the associations of BaPWV with TC^10,11^ and systolic blood pressure (SBP)^12–18^. Our previous study^12^ showed that the increase in arterial stiffness might precede the increase in blood pressure, and each 1-unit increase in BaPWV was associated with an increase of SBP by 0.09 mmHg. However, no study has explored whether the TC level increases the SBP by affecting BaPWV. In addition, the degree of mediation associated with the elevation of BaPWV is uncertain. In the current study, we performed a mediation analysis in a large community cohort of 45,092 adults to investigate the relationships among TC, arterial stiffness, and SBP and to quantify the effect of the TC level attributed to changes in BaPWV on SBP.

## Results

### General information

**Figure 1:**
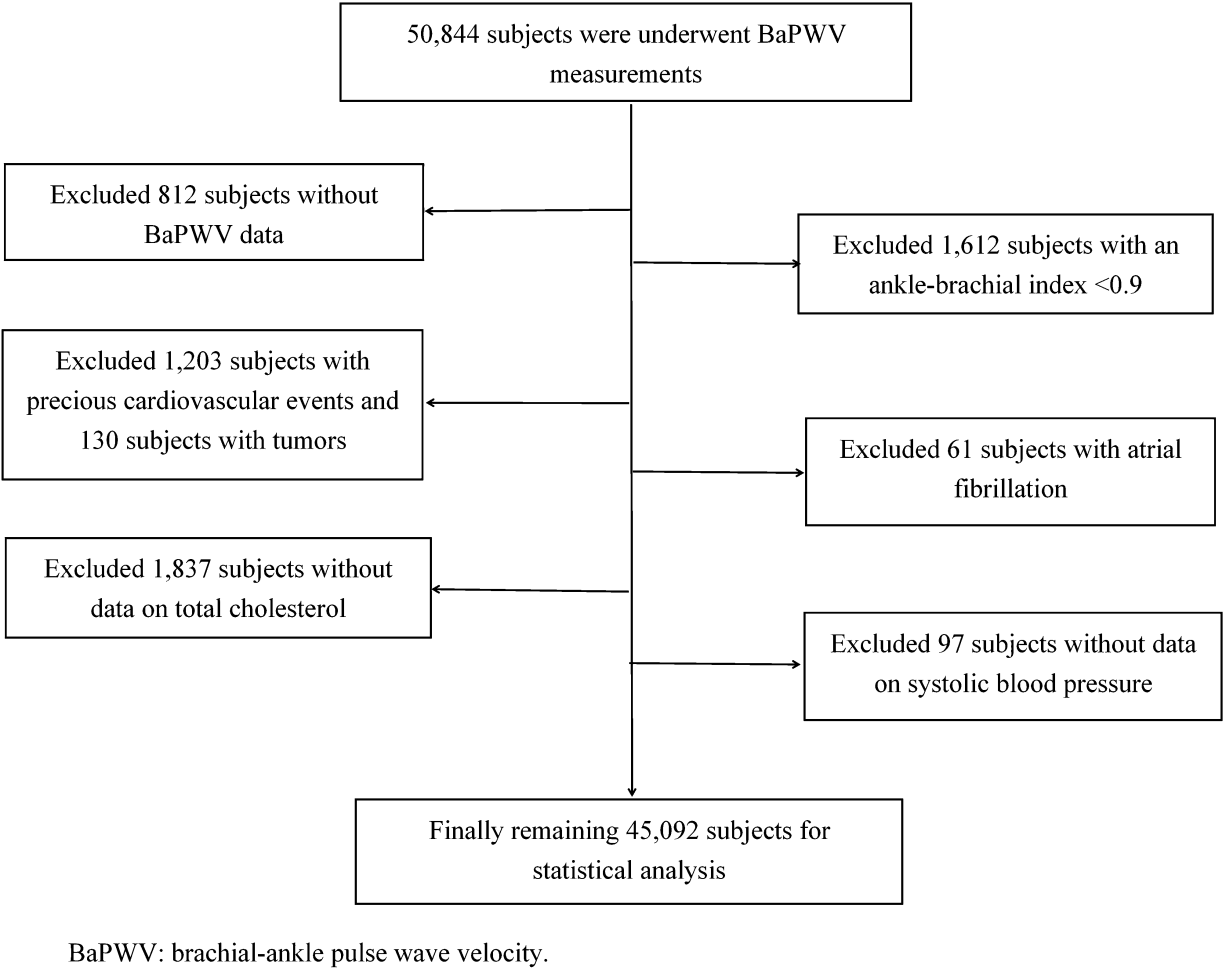
Inclusion/exclusion flowchart for study participants.

BaPWV measurements were carried out totally on 50,844 participants, among whom 812 were excluded because of a lack of BaPWV data, 1612 because of ankle-brachial index of < 0.9, 1203 because of cardiocerebrovascular events, 130 because of tumors, 61 because of atrial fibrillation, and 1837 because of a lack of TC data. After additional participants with a lack of SBP data were excluded, 45,092 participants entered the final analysis (Fig. 1). These participants comprised 28,371 males (62.9%) and 16,721 females (37.1%), with a mean age of 54.04 ± 13.09 years. Compared with the females, the males had a significantly higher age, TC level, SBP, BaPWV, fasting blood glucose level, heart rate, waist circumference, and the proportions of alcohol consumption, smoking, physical exercise, antihypertensive therapy and glucose-lowering treatment, which was of statistical significance(all *P* < 0.05, Table 1).

**Table 1:** Characteristics of the study population gender.

Characteristics	Female N = 16,721	Male N = 28,371	*P* value
Age, y, Mean ± SD	53.48 ± 12.98	54.36 ± 13.15	< 0.01
Total cholesterol, umol/L, Mean ± SD	4.96 ± 1.74	5.05 ± 1.58	< 0.01
Systolic blood pressure, mmHg, Mean ± SD	131.21 ± 21.80	144.42 ± 19.24	< 0.01
BaPWV, cm/s, Mean ± SD	1344 ± 299	1502 ± 280	< 0.01
Fasting blood glucose, mmol/L, Mean ± SD	5.31 ± 1.13	5.66 ± 1.43	< 0.01
Heart rate, bpm, Mean ± SD	72.92 ± 7.40	73.12 ± 8.19	0.04
Waist circumference, cm, Mean ± SD	79.81 ± 8.30	87.85 ± 7.94	< 0.01
Alcohol consumption, n (%)	1576 (9.5%)	16,306 (57.5%)	< 0.01
Smoking, n (%)	197 (1.2%)	13,796 (48.6%)	< 0.01
Physical exercise, n (%)	1776 (10.7%)	3935 (13.9%)	< 0.01
Antihypertensive therapy, n (%)	2518 (15.1%)	5879 (20.5%)	< 0.01
Glucose-lowering treatment, n (%)	1012 (6.0%)	2143 (7.5%)	< 0.01

*BaPWV* brachial-ankle pulse wave velocity, *SD* standard deviation.

### Associations among TC, BaPWV, and SBP

With TC and BaPWV as the dependent variables and SBP as the independent variable, the analysis showed that both TC and BaPWV were positively correlated with SBP. After the relevant variables were adjusted, SBP increased by 0.33 mmHg and 0.044 mmHg, respectively, for every one standard deviation increase in TC and BaPWV (both *P* < 0.05). With TC as the dependent variable and BaPWV as the independent variable, the analysis showed that TC was positively correlated with BaPWV, and BaPWV increased by 5.34 cm/s for every one standard deviation increase in TC (*P* < 0.05). In both the male and female subgroups, both TC and BaPWV were positively correlated with SBP, and TC was positively correlated with BaPWV, as in the total cohort (Table 2).

**Table 2:** Associations among TC, BaPWV, and SBP.

Parameters	total		female		male	
	β	*P* value	β	*P* value	β	*P* value
TC-SBP	0.33	< 0.01	0.89	< 0.01	0.32	< 0.01
BaPWV-SBP	0.044	< 0.01	0.044	< 0.01	0.043	< 0.01
TC-BaPWV	5.34	< 0.01	9.33	< 0.01	4.42	< 0.01

Adjust age, gender (in total), waist circumference, fasting blood glucose, physical exercise, smoking, alcohol consumption, heart rate, antihypertension, glucose-lowering treatment.

*TC* total cholesterol, *BaPWV* brachial-ankle pulse wave velocity, *SBP* systolic blood pressure.

### BaPWV mediated the association between TC and SBP in the mediation analysis

We performed a mediation analysis to better understand the relationships of SBP with TC and BaPWV. The mediation analysis showed that the total effect of the TC-induced SBP elevation was 1.34 in the whole cohort, among which the increase in SBP contributed to TC was mediated by arterial stiffness in more than half of the total effect (indirect effect, 0.73; percent mediated, 54.5%). The total effect of TC-induced SBP elevation was 1.46 in the males, which was slightly higher than that in the whole cohort, among which the increase in SBP contributed to TC was mediated by arterial stiffness in less than half of the total effect (indirect effect, 0.70; percent mediated, 47.9%). Although TC still caused an increase in SBP in the females, its total effect, direct effect, and indirect effect were not statistically significant (Table 3).

**Table 3:** Mediation effect of BaPWV in the association between TC levels and SBP.
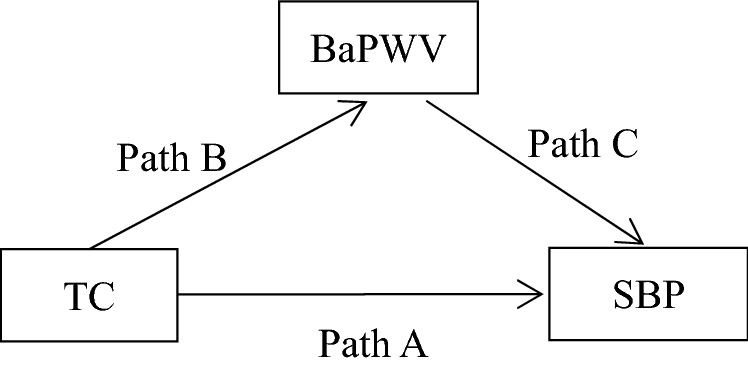

Group		Total effect	Direct effect (Path A)	Indirect effect (Path BC)	Percent mediation (%)
Model 1	Total	1.60 (1.47–1.73)	0.21 (0.11–0.31)	1.39 (1.10–1.81)	86.9
	Female	1.61 (1.31–1.90)	0.091 (− 0.13–0.31)	1.52 (0.70–4.13)	94.4
	Male	1.68 (1.47–1.90)	0.50 (0.32–0.68)	1.18 (1.00–1.36)	70.2
Model 2	Total	1.34 (0.85–1.83)	0.60 (0.20–1.00)	0.73 (0.43–1.01)	54.5
	Female	0.57 (− 0.41–1.52)	0.041 (− 0.71–0.84)	0.52 (− 0.14–1.20)	91.2
	Male	1.46 (0.88–1.97)	0.75 (0.28–1.22)	0.70 (0.41–1.03)	47.9

Model 1: Unadjust.

Model 2: Adjust age, gender (in total), waist circumference, fasting blood glucose, physical exercise, smoking, alcohol consumption, heart rate, antihypertension, glucose-lowering treatment.

## Discussion

In this large community-based study of 45,092 participants, we found that both TC and BaPWV were significantly associated with SBP and that TC was associated with BaPWV. After the relevant variables were adjusted, SBP increased by 0.33 mmHg and 0.044 mmHg, respectively, for every one standard deviation increase in TC and BaPWV; for every one standard deviation increase in TC, BaPWV increased by 5.34 cm/s. Our findings are consistent with the results of previous studies^9–17,19^. Mancia^19^ evaluated 2045 participants and found that SBP increased by 0.27 mmHg for every 1-unit increase in TC. However, the correlation of arterial stiffness with SBP and TC remains controversial^14,15,20,21^, and no single study has elucidated the cross-sectional relationships among TC, BaPWV, and SBP.

The mediation analysis in the current study demonstrated the correlations among TC, BaPWV, and SBP. The analysis also considered the effects of TC and BaPWV on SBP. It was found that half of the TC-induced SBP elevations were mediated by TC-induced arterial stiffness. Such a mediation effect was slightly lower in males but was not statistically significant in females. To the best of our knowledge, no previous study has investigated the role of TC in inducing SBP elevation by affecting BaPWV. However, previous studies have shown that the development of arterial stiffness is the main cause of blood pressure changes and ultimately promotes the development of systolic hypertension and that blood lipids play an important role in the occurrence and development of arterial stiffness^12,22^. In addition, studies have suggested that the effect of hypercholesterolemia on blood pressure may be related to the effect of a high cholesterol level on arterial stiffness^8,23^. However, these studies only presented a hypothesis, and to what extent arterial stiffness exerts its mediation effect remains unclear. The present study showed that TC-induced SBP elevation was not mediated by TC-induced arterial stiffness in females, which may be explained by the relatively lower blood lipid levels, younger ages, and fewer risk factors for arterial stiffness and hypertension in females than males. Our previous study demonstrated the effect of age on arterial stiffness and blood pressure^12^; specifically, the elevated blood pressure contributed to arterial stiffness was more pronounced in participants of advanced age. Previous studies have shown that the effects of high blood glucose and smoking on arterial stiffness and blood pressure should not be ignored^24–26^.

The possible explanations for the findings in the current study are as follows. First, TC is known to be harmful to endothelial function, and an elevated serum TC level leads to arterial stiffness by increasing the vascular smooth muscle cell response to angiotensin II and reducing nitric oxide bioavailability^27,28^. These pathophysiological pathways are consistent with the findings of our current study, which the TC level is associated with SBP elevation. Second, the oxidized lipids accumulate along with the inflammatory reaction and migrate to the tunica intima, causing degradation of collagen, elastic fibers and proliferation of smooth muscle cells, thus leading to the development of arterial stiffness^29–31^. Third, blood lipid can lead to accumulative plaque, which narrows the artery, aggravate arteriosclerosis and eventually causes the increase of systolic blood pressure^32,33^.

Given the close relationships of TC, arterial stiffness, and hypertension with cardiovascular disease^34–36^, the incidence of hyperlipidemia continues to increase^37^, and the cardiovascular disease burden associated with dyslipidemia will likewise continue to rise. There is evidence that arterial stiffness may be reversed or improved by lifestyle changes such as aerobic exercise and weight reduction^38,39^. We found that TC and arterial stiffness were risk factors for elevated blood pressure, and arterial stiffness mediated the relationship between hyperlipidemia and blood pressure. Therefore, we suggest that clinicians may delay or improve arterial stiffness and elevated blood pressure by controlling blood lipids, especially in patients with hypertension and hyperlipidemia, which may help to further prevent cardiovascular disease. For patients with hyperlipidemia, however, clinicians should pay attention to the development of arterial stiffness and changes in blood pressure when using statins. If necessary, a treatment strategy to prevent or reverse aortic stiffness should be used, which may help to prevent the occurrence of hypertension and control blood pressure.

This study had certain limitations. First, the study population was limited to Kailuan Group employees, most of whom live in North China. Thus, whether the findings of this study can be applied to other populations remains uncertain. However, the homogeneity of the study cohort could reduce potential biases; furthermore, the large sample size increased the scalability of our findings for the Chinese population. Second, BaPWV rather than carotid–femoral pulse wave velocity, the gold standard for arterial stiffness, was used as the indicator for arterial stiffness in the current study. However, previous studies have shown that BaPWV can be used to assess the degree of arterial stiffness in a simple, repetitive, and noninvasive manner. Moreover, there was a strong correlation between BaPWV and carotid-femoral pulse wave velocity (correlation coefficient of 0.73), suggesting that BaPWV can be used to measure arterial stiffness^40,41^. In addition, previous studies have also suggested that BaPWV could serve as an alternative to carotid-femoral pulse wave velocity due to the following two reasons. One is that BaPWV has stronger association with age and blood pressure, the other is that BaPWV is of normal range and frequently applied in Asian countries^42^. Third, we did not adjust for statin therapy. Previous studies have shown that statin therapy can affect blood pressure changes by affecting blood lipid levels^43^. However, statin therapy has an impact on TC, BaPWV, and SBP, it is difficult to evaluate the influence of statin therapy on the outcome of mediation analysis. In our future studies, we will pay special attention to the effects of statins. Fourth, there is an interval between the measurement of TC and BaPWV, which may lead to certain error in our result. However, this interval was short and its median value was 0.92 year. Finally, previous studies have shown that hyperglycemia is an important factor affecting arterial stiffness and vascular changes^24^. Although we only adjusted for fasting blood glucose in this study, fasting blood glucose alone is not sufficient for detecting blood glucose problems. The oral glucose tolerance test should be used in future studies.

In conclusion, TC is positively correlated with BaPWV and SBP, whereas BaPWV is positively correlated with SBP. Almost half of the increase in SBP contributed to TC is mediated by arterial stiffness.

## Methods

### Sarticipants

The Kailuan Study (registration number: CHiCTR-TNC-1100 1489) is a prospective longitudinal cohort study^44–46^. Beginning in 2006–2008, health check-ups were carried out for the in-service and retired employees of Kailuan Group in 11 hospitals including Kailuan General Hospital and its branches every 2 years, during which the results of anthropometric measurements and biochemical tests were collected. The medical staff who participated in the first session of health check-ups performed the subsequent physical examination sessions for the same population at the same site using the same protocol (including the survey content, physical examinations, and laboratory tests). Measurements of BaPWV were performed beginning in 2010. The health checkup data were entered by the assigned personnel and summarized by Kailuan General Hospital. The study was conducted in accordance with the Declaration of Helsinki, and the protocol was approved by Kailuan General Hospital. All participants provided written informed consent.

The inclusion criteria were participation in the health check-ups from 2010 to 2016 and agreement to participate in the present study with provision of written informed consent. The exclusion criteria were missing data on TC, SBP, or BaPWV; a history of cardiocerebrovascular events and/or tumors; and an ankle-brachial index of < 0.9.

The methods and results of the epidemiological surveys, anthropometric measurements, and biochemical tests have been previously published^44,47–49^.

### Laboratory tests

The participants were fasted for at least 8 h, and 5 ml of fasting elbow venous blood was then collected into an EDTA vacuum tube at 7:00 to 9:00 am on the day of the health check-up. After centrifugation at room temperature (24 °C) at 3000 rpm for 10 min, the upper layer was harvested for detection within 4 h. The TC and fasting blood glucose levels were measured after centrifugation. TC was measured with the enzymatic method (Mind Bioengineering Co. Ltd, Shanghai, China), and fasting blood glucose was measured by the hexokinase method (7600 Auto matic Analyzer, Hitachi, Tokyo, Japan)^49^.

### Blood pressure measurements

Before blood pressure measurement, the participants were asked not to smoke or drink tea or coffee within 30 min. The participants were instructed to empty the bladder, keep their mood stable, and rest on a chair for 15 min. The blood pressure was measured with the subject in a sitting position in a comfortable environment. A table with its height suitable for placement of the participants’ arms was used. The right upper limb was placed at 45 degrees abduction, with the elbow positioned at the same level as the heart. Cuffs suitable for the participants’ upper arms were used (12 × 22 cm for arm circumference of 22–26 cm, 16 × 30 cm for arm circumference of 27–34 cm, 16 × 36 cm for arm circumference of 35–44 cm, and 16 × 42 cm for arm circumference of 45–52 cm). The cuff was evenly and tightly attached to the skin and wrapped around the upper arm, with the lower edge about 2.5 cm above the elbow and the center of the cuff located above the brachial artery. The right radial artery blood pressure was measured using a calibrated mercury sphygmomanometer. The deflation was even and slow, with a decline rate of 2–4 mmHg/beat. The SBP was read at the first appearance of Korotkoff sounds, with the readings accurate to 2 mmHg. The participants’ blood pressure was measured three times at an interval of 1 min during each health check-up session. The average of these three blood pressure measurements was used as the final SBP value.

### BaPWV measurement

BaPWV^49^ was measured using an arteriosclerosis detection device (Omron Health & Medical (China) Co., Ltd., BP-203 RPE III). The temperature of the examination room was kept within 22–25 °C. The subject was asked not to smoke or drink tea or coffee before the measurement. After the subject had rested for more than 5 min, basic data including gender, age, height, and weight were recorded. The subject was then asked to wear a thin coat. At the beginning of the measurement, the subject remained quiet. The pillow was removed, and the subject lay in the prone position. Both hands were placed on either side of the body, with palms facing upward. The blood pressure cuffs of the four limbs were tied to the upper arms and the ankles. The cuffs were placed over the bare upper arms with the artery mark positioned directly over the brachial artery, and the lower edge of the cuff was about 2–3 cm away from the antecubital fossa. The marker of the lower limb cuff was located on the inner side of the lower limb, with the lower edge of the cuff about 1–2 cm away from the medial malleolus. The heart sound collection device was placed in the participants’ precordial region, and the left and right wrists were connected with clip leads. The measurements were repeated twice for each subject, and the second measurement data were used as the final result. The BaPWV was measured on both the left and right sides, and the larger measured value was used for analysis. It was difficult for us to collect BaPWV of all the participants in such a short time because of the large sample size. There has been also no relevant stipulation for the intervals between the indicators in Mediation Analysis. As a result, we have chosen the TC which was measured before and closest to the BaPWV as the indicator (the interval range: 0.02–8.42 year, median interval: 0.92 year, mean interval: 0.91 year ).

### Statistical methods

Statistical analysis was performed with the SAS 9.4 software package (SAS Institute, Cary, NC, USA). Continuous variables are presented as mean ± standard deviation, and comparisons between the two genders were performed using one-way analysis of variance. Categorical variables are presented as n (%), and inter-group comparisons were based on the chi-square test. The correlations among TC, SBP, and BaPWV were analyzed by multivariate linear regression models. Mediation analysis was performed to determine whether the effect of TC on SBP can be explained by arterial stiffness. A mediation analysis is used to test how a given mediator (BaPWV) affects the relationship between an independent variable (TC) and an outcome variable (SBP), quantifying the overall effect (the association between the independent and outcome variables), the direct effect (the overall effect unaffected by the mediator), and the indirect effect (the effect of the independent variable on the outcome variable attributed to the mediator). The proportion of the effect is then calculated by dividing the indirect effect by the total effect, which represents the proportion of the total effect attributable to the mediator. To reduce the impact of gender on the analysis results, the participants were divided into two subgroups based on gender. A *P* value of < 0.05 was considered statistically significant. All analyses were two-tailed.

## Data Availability

Because of data protection, datasets generated and analyzed during the current study are not published, but the appropriate authors may have access to and/or analyze the datasets from the current study if reasonably required.

## Abbreviations

BaPWV: Brachial-ankle pulse wave velocity
SBP: Systolic blood pressure
TC: Total cholesterol
